# A consensus layer V pyramidal neuron can sustain interpulse-interval coding

**DOI:** 10.1371/journal.pone.0180839

**Published:** 2017-07-13

**Authors:** Chandan Singh, William B. Levy

**Affiliations:** Departments of Neurosurgery and of Psychology, University of Virginia, Charlottesville, VA, United States of America; University of Sussex, UNITED KINGDOM

## Abstract

In terms of a single neuron’s long-distance communication, interpulse intervals (IPIs) are an attractive alternative to rate and binary codes. As a proxy for an IPI, a neuron’s time-to-spike can be found in the biophysical and experimental intracellular literature. Using the current, consensus layer V pyramidal neuron, the present study examines the feasibility of IPI-coding and examines the noise sources that limit the information rate of such an encoding. In descending order of importance, the noise sources are (i) synaptic variability, (ii) sodium channel shot-noise, followed by (iii) thermal noise. The biophysical simulations allow the calculation of mutual information, which is about 3.0 bits/spike. More importantly, while, by any conventional definition, the biophysical model is highly nonlinear, the underlying function that relates input intensity to the defined output variable is linear. When one assumes the perspective of a neuron coding via first hitting-time, this result justifies a pervasive and simplifying assumption of computational modelers—that a class of cortical neurons can be treated as linearly additive, computational devices.

## Introduction

This study addresses three contemporary topics that are part of understanding neural computation and neural codes: (1) Is there a cortical neuron whose output is consistent with the assumption of linear additivity? (2) What are the relative contributions of the stochastic processes that limit information flow through a neuron? And (3) what is the mutual information, bits-per-spike, for such a neocortical pyramidal cell?

McCulloch and Pitts [1] introduce the computational neuron as a deterministic threshold-linear device. Gerstein and Mandelbrot [2] consider a linearly additive neuron in a stochastic setting (their “random walk model”). However, even in this novel work, which assumes a linearly additive neuron, there is the absence of whole-hearted support for such additivity because of a neuron’s known leak-currents. Indeed, although there has been some biophysical modeling that attempts to justify a linear neuron [3–5], much more effort has gone into understanding the stochastic, leaky-neuron model [6–13]. The focus here is motivated by interpulse interval (IPI) coding since it has the highest bit-rate possible of any code using constant-amplitude pulses [14–17] (proof: if there is no information in amplitude, all information must be in interpulse intervals. A code that retains the value of every interpulse interval loses no information). Moreover, for fixed-amplitude pulses and fixed and axonal leak rates that are suitably more expensive than a pulse, no code is more energy efficient in terms of bits / joule than IPI coding.

Any spike-generating neuron is, fundamentally, non-linear with much research aimed at understanding such non-linear behavior. Such research includes identifying differential equations that reproduce observed firing patterns (e.g., [18–20]). Our perspective asks a different question: Is there a cortical neuron whose output—time-to-spike (TTS) from rest to spike generation—is consistent with the assumption of linear additivity of synaptic excitation? By linear additivity we mean that the TTS is inversely directly proportional to the synaptic intensity. To achieve such linearity clearly requires an underlying nonlinear model since both the RC-leak of a neuron and the driving voltage of synaptic excitation cause sublinearity of the net synaptic effects.

Beginning with certain empirical studies, there is progressively stronger motivation for considering a physiological, functioning pyramidal neuron of the cortex to be a linearly additive computational device. The work of Ferster [21–24] and follow-up research [25–27] stands out in this regard because of the use of intracellular recording in perceptually relevant situations. Other intracellular work supports the existence of linearly additive voltage-ranges [28–31]. However, it is easy to find references to non-linear behavior that includes sublinear and supralinear [31, 32]. But in choosing one’s motivation, we come down on the side of intracellular observations in awake behaving organisms as the greatest relevance.

Beyond such empirical literature, there is the computational and code-theoretic literature that implicitly assume some kind of linear excitation function. The two, non-mutually exclusive positions are a population-coding conjecture [33–39] and a single neuron Bayesian computational conjecture [40–45]. Implicitly or explicitly, such theoretical stances assume that a neuron’s appropriate range of operation is characterized by linear additivity of its synaptic input events.

Running against this trend for linear additivity, there is a long history of biophysical analyses beginning with Stein [12] and continuing up to the present day, with more complicated models (e.g. the Hodgkin-Huxley model of [13] and the QIF model of [20]). In purely passive models, a neuron’s behavior between resting potential and spike-threshold is subadditive in excitation. In models with leak and voltage-dependency, there can be subadditivity, superadditivity, or both. In any case, there is a path dependency (voltage as a function of time); this dependency prevents the exact inversion of first hitting-time (time to reach threshold) into input intensity. That is, the first hitting-time for an RC-leaky neuron, or more complicated models built on top of an RC-leaky neuron, cannot be properly inverted into the average intensity of net synaptic excitation for any one, specific IPI; although one could claim the average path is good enough, we do not pursue this option as it surely loses information.

The focus here is on one particular recent biophysical model culminating in [46]; this model reproduces action potential shape and firing found in empirical studies. This model of a layer V pyramidal neuron of cerebral cortex is published as non-stochastic and is selected because it seems to be the “consensus pyramidal neuron” with several labs using the same model morphology [19, 47–49] and not infrequently the same voltage-activated conductances (VGCs) [50], and even some agreeing in terms of the varieties and placements of VGCs [46, 49, 51, 52]. The VGCs present in these models include, two voltage-controlled K-channels (a delayed rectifier and a K_A_-type) and two types of voltage-controlled Na-channels (Nav 1.2 and Nav 1.6) with a specific microscopic distribution, notably a high concentration of the low-threshold Nav 1.6 channels in the axonal initial segment (AIS) [46, 52–55].

Here, the deterministic model (continuous Na-channel conductances as a function of voltage) is the starting point. For both deterministic and stochastic intensities, the relationship between injected current and TTS is quantified by relating input intensity to inverse TTS. The results suggest that, for the neocortical neuron studied here whose underlying biophysics are non-linear, a linearly additive model well-approximates the input-output behavior over a range of excitatory intensities. Using the inverse linear relationship for decoding, the model is then used to evaluate noise or noise-like effects. Three noise or noise-like processes are considered—thermal, Na-channel shots, and random synaptic arrivals; synaptic arrivals are, by far, the dominant noise-source. Here, Shannon’s mutual information measures information transmission; the channel input is a randomly chosen, scalar latent variable that parameterizes the Poisson intensity of the totality of the synaptic activations, and the output variable is 1/TTS. The estimate of this mutual information is approximately 3.0 bits per spike.

## Results

The results here begin with examples that show inverse TSS is linearly related to input intensity. This inverse linearization motivates the assumption of an inverse Gaussian, first hitting-time probability-distribution. This hypothesis leads to (i) an examination of the statistical distribution of TTS and (ii) a comparison of the contribution of three physical sources of randomization that affect the variance of this first-hitting time distribution. Finally, with the assumption of a prior distribution, mutual information of a defined neural computation can be calculated.

### Linearized inverse time-to-spike

**Fig 1:**
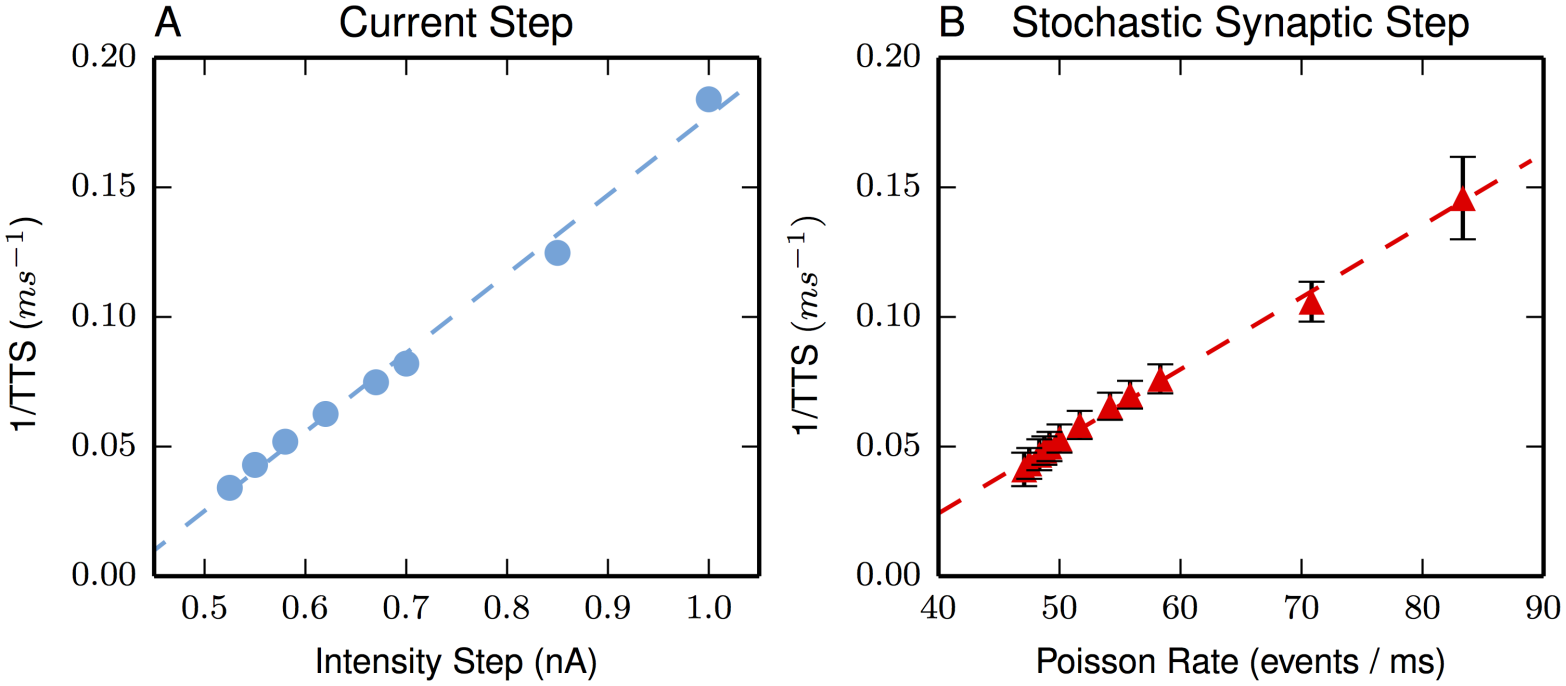
Inverse TTS approximates a linear function of excitation. Excitation is either (A) a point dendritic current-step or (B) a spatially dispersed, synaptic activation. Lines are best linear fits (see text). Each point is an average of 120 excitations from rest. The error bars (SEM) for the current-step are within the plot points. All points but the highest intensities always had spike initiation at the AIS. At the largest intensity on each curve, the spike originated in the dendrite 20 percent of the time.

The model sustains an inverse linear range for current-steps ranging from 0.51 nA to 0.85 nA when there is the requirement that action potential initiation begins at the AIS; relaxing this last requirement slightly extends the range (the upper bound becomes 1.0 nA). Fig 1 illustrates this inverse relationship. Fig 1A uses the point-injection of a current-step. the slope is 0.30 μC^-1^ with an extrapolated y-intercept at zero-current of -0.13 ms^-1^. Spatially-distributed, synaptic activation (with an input intensity rate λ) also has the linear relationship; here, the slope is 0.0028 events^-1^ with a zero-intensity intercept of -0.09 ms^-1^. Perhaps due to the spatially distributed nature of synaptic activation, the currents required to induce a spike are consistently larger than the currents delivered by point-current injections. For example, the rate of synaptic activation needed to achieve a 1 nA spatially distributed current injection is 83.3 events/ms (assuming a 50 mV driving potential, 200 pS / synapse, and a synaptic event duration of 1.2 ms). By comparison, for the same net current injection, 1/TTS for the point injection is 0.18 while it is 0.14 for the spatially distributed synaptic current.

### Variation in TTS

The overriding variation of the TTS using synaptic activation arises from the variability of the stochastic process itself. This conclusion is best seen by comparing the deterministically generated TTSs against the stochastic synaptically generated TTSs, both with stochastic sodium channels.

**Fig 2:**
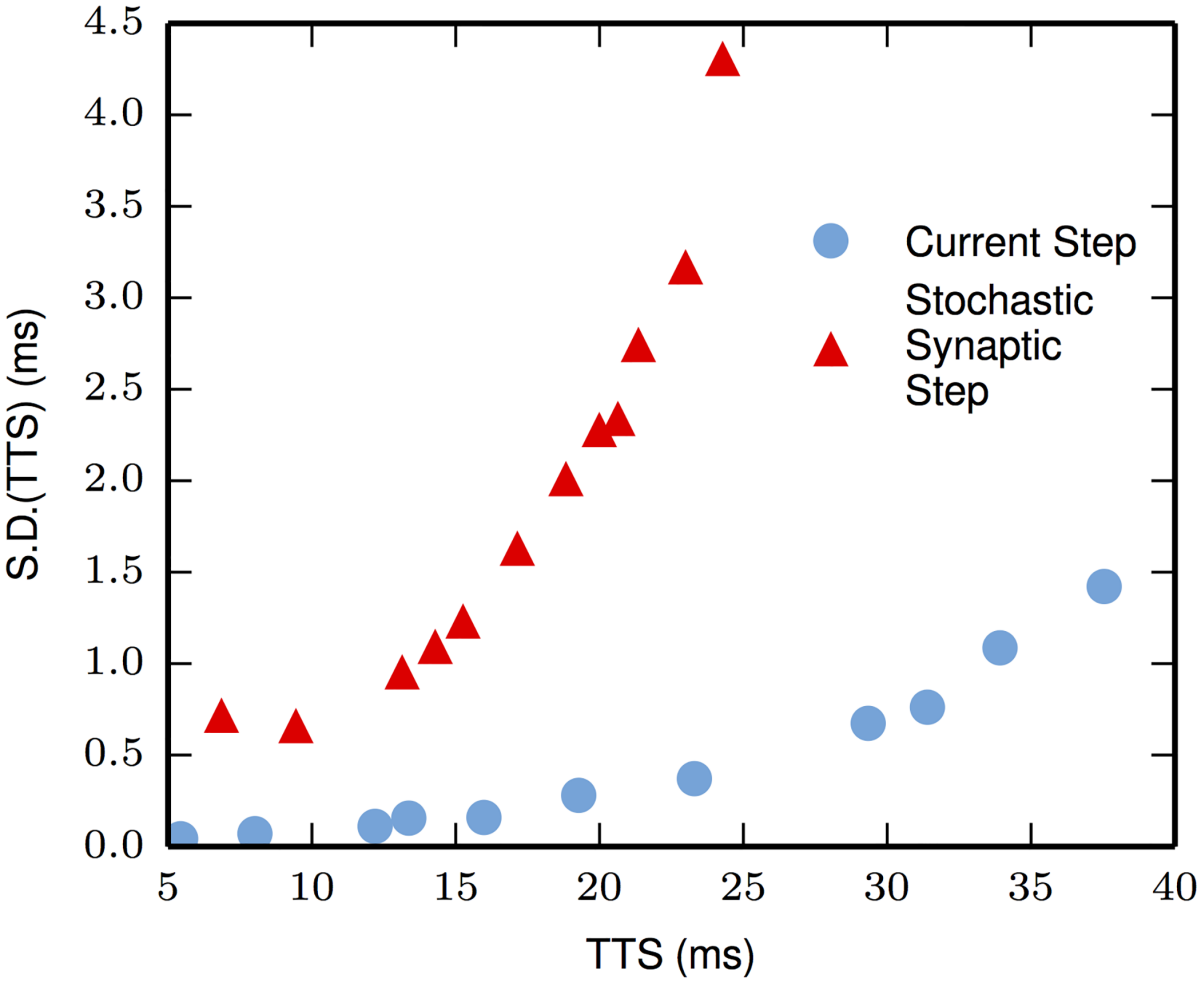
Synaptic activation increases variation. The only variation in TTS using a current-step is due to the stochastic nature of Na-channel activation. Random synaptic activation greatly increases the variation in TTS. Plot points, left-to-right, correspond to the reverse-ordered successive intensities of Fig 1.

For the highest intensities, the ratio of the variances is more than 200, while for the lowest intensities the ratio of is the variances is more than 40. The standard deviations are plotted in Fig 2 for better visualization of the size of this effect as a function of inverse intensity (note that when viewing these curves, the leftmost values of TTS correspond to the highest intensities). The sodium channel shot-noise increases as the TTS increases; this shot-noise increase is mainly due to the number of events needed to fire a spike; i.e., threshold rises as TTS increases. Such a requirement for a greater amount of depolarization means that more sodium channels need to be activated to evoke an action potential. This larger number of sodium channels correlates with a larger standard deviation of TTS. Nevertheless, there is no spontaneous firing in this neuron; the contribution of an individual channel shot is not enough to fire the neuron until the neuron is very close to threshold.

As is well-known for additive point processes, a larger number of events with smaller values (conductances) leads to lower average variance of the steady-state voltage. This lower variance in the voltage is reflected as lower variance in the TTS. A simple model of this relationship between steady-state variance of the voltage and the variance of the TTS (as a first hitting-time distribution) is seen in the standard result [56] that the variances of a first hitting-time and of a Brownian motion (which shot noises can approximate) are proportional.

**Fig 3:**
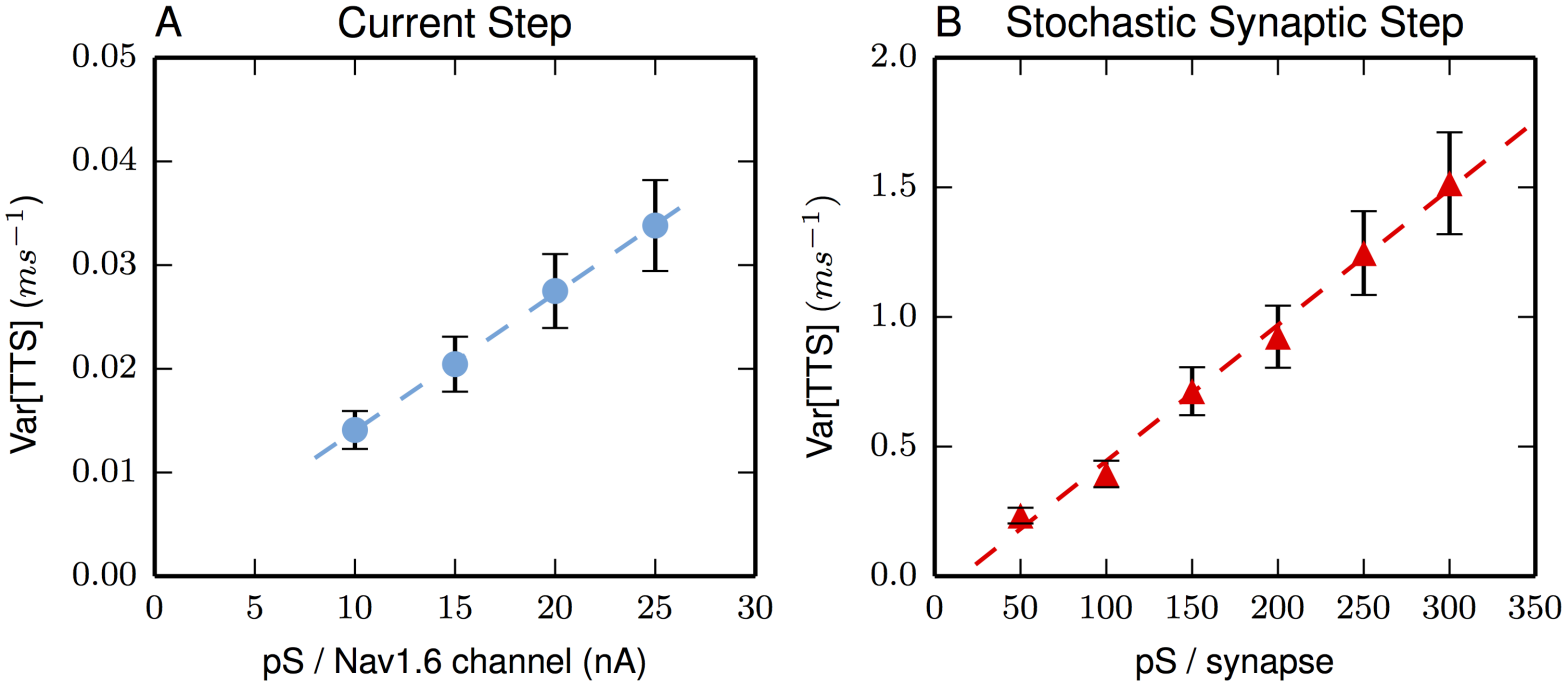
Synaptic shot-noise far exceeds Na-channel shot-noise. Random synaptic activation greatly increases the variation in TTS. (A) The only variation in TTS using a current-step is due to the stochastic nature of Na-channel activation. TTS variance increases as individual Na-channel conductance events get larger while keeping $\bar{g}$ constant. By comparison in (B), the synaptic conductance events create much more variance. Note the y-axis scale differences. A current-step of 0.7 nA generates the data of (A). In (B), stochastic activation for each point is on average the same with a total conductance of 16.6 nS. Error bars are SEM. Lines are best linear fits (see text).

As one would expect, maintaining a constant total conductance ($\bar{g}$) while changing the pS/event changes the variance. In fact, there is a linear relationship between the size of the individual conductance event and the variance (see Fig 3). The best linear fit for Fig 3A is Var[TTS] = 0.0012*x + 0.003 with an *R*^2^ value of 0.984 and the fit for Fig 3B is Var[TTS] = 0.0052*x – 0.0797 with an *R*^2^ value of 0.993. The conductance of a sodium channel is in the vicinity of 10 – 20 pS [57–59], while the conductance of a synapse might average around 200 pS. Synaptic noise dominates, even when varying the parameters of the model (*e*.*g*. pS/channel, pS/synapse). When combining both sources of noise, the total noise is essentially indistinguishable from the noise generated solely by the synaptic input.

**Fig 4:**
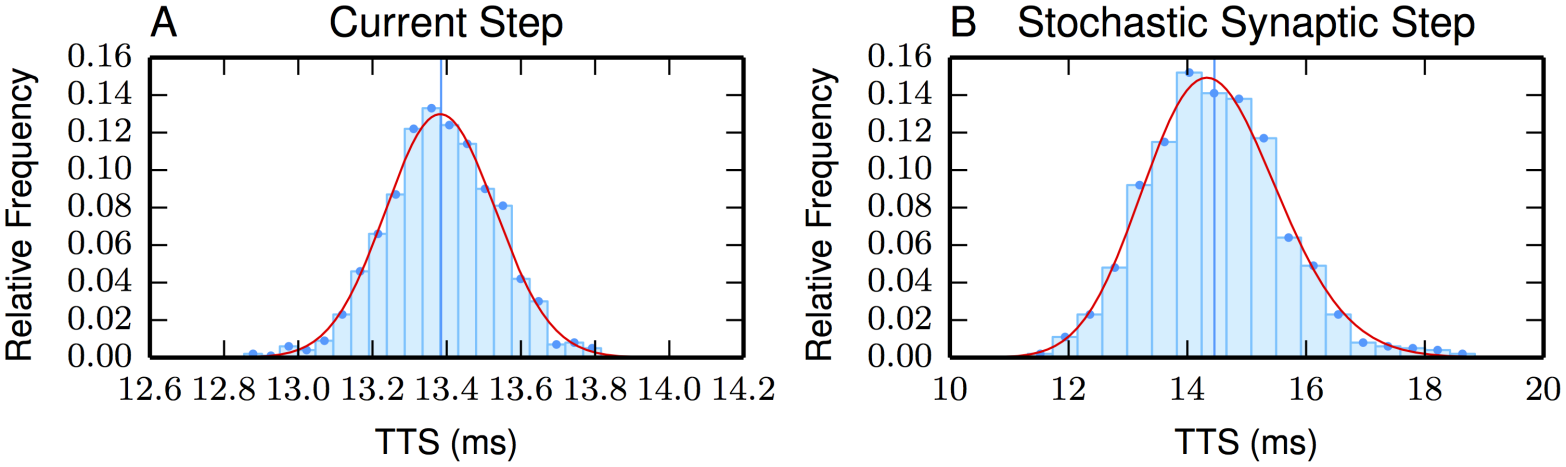
TTS relative frequency histogram and overlaid inverse Gaussian distribution with the same mean and variance. (A) is generated by a current-step of 0.67 nA, the mean TTS is 13.38 ms (vertical line) and the variance is 0.022 ms^2^. (B) is generated by Poisson synaptic activation (λ = 55.8 events/ms), the mean TTS is 14.46 ms (vertical line) and the variance is 1.25 ms^2^. One thousand simulations produce each of the histograms. Current and synaptic activations begin at TTS = 0. Notice the x-axis scale difference.

Finally, as will be important in the next section, it is necessary to have reliable probability distributions for the relationship between intensity and TTS. Fig 4 again shows that synaptic variation swamps sodium channel-induced variation (whatever noise is produced in the current-step histogram in Fig 4A will be biophysically added into the stochastic synaptic step histogram in Fig 4B). However, more to the point is that the TTS distribution can be fit by an inverse Gaussian distribution (see Fig 4).

### Thermal noise

**Fig 5:**
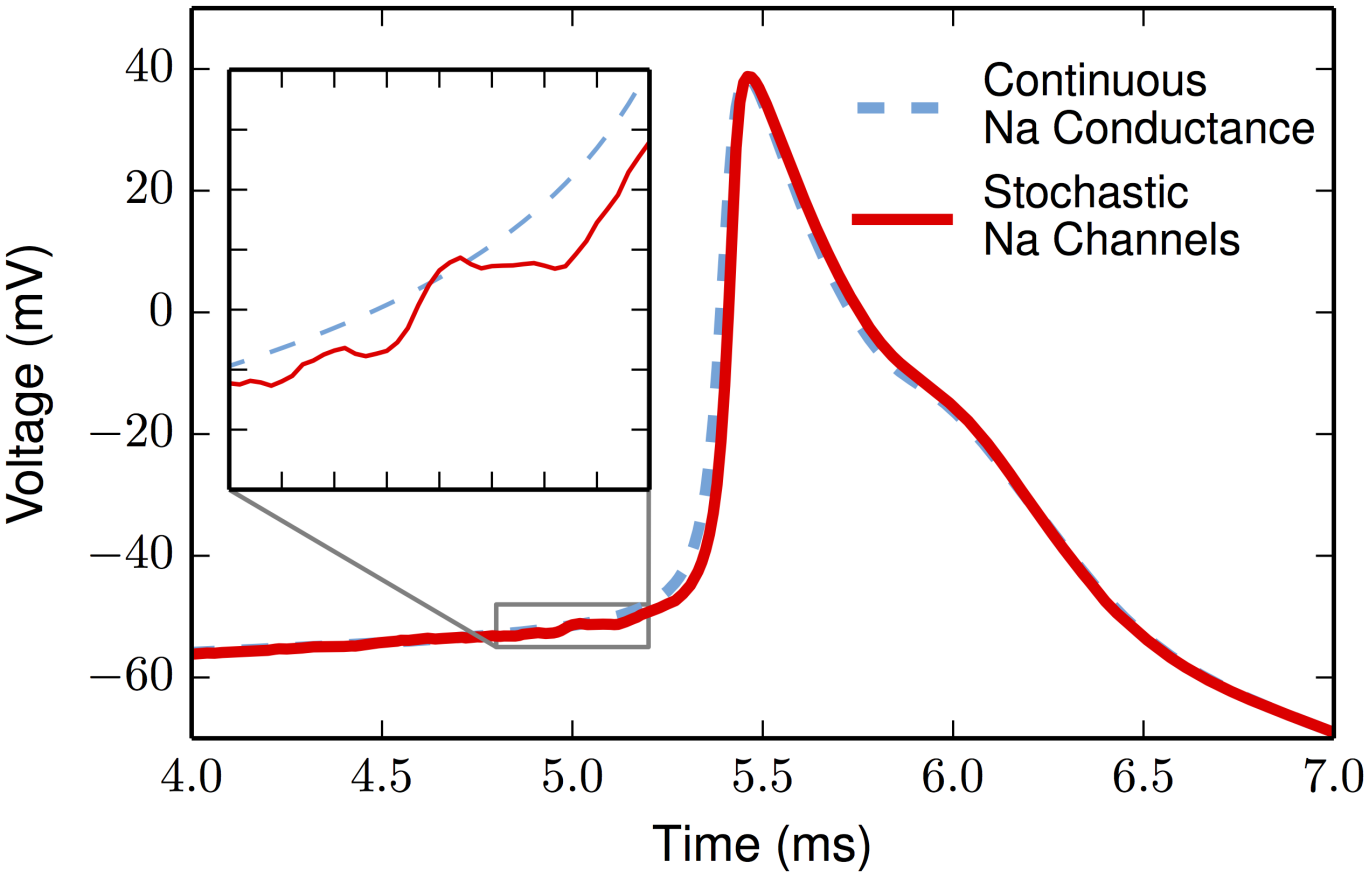
Comparison of a stochastic- and a deterministic-based action potential. The deterministic action potential (blue dashed line) reproduces the result of Hu et al; their action potential initiates at the AIS and spreads to the soma and apical dendrite. Aligned, peaked to peak, is a second action potential (solid red line) using stochastic Na-channels (both Nav 1.2 and Nav 1.6). Both action potentials are generated by the same somatic current-step of 1 nA. Inset y-axis goes from -55 mV to -48 mV (increments of 1 mV); inset x-axis goes from 4.8 ms to 5.2 ms (increments of.05 ms).

Thermal noise is also present, but it is the noise source of least concern. Although thermal noise increases with increasing resistance, the low-pass property of a resistive-capacitive circuit when recording across the capacitor exactly cancels out the resistance effect by lowering the high-frequency cutoff of the filter [60]. Thus, the thermal noise (calculated as the expected value of the variance of the voltage) is equal to *kT*/*C* = 1.78 x 10^-11^ V^2^ (C is the capacitance of the neuron, 240 pF; *k* is the Boltzmann constant; and T is body temperature, 310 K). Thus, the standard deviation of this zero-centered noise is 4.22 μV. Compare this value to the shot noise fluctuations shown in Fig 5 inset; it is much smaller than the sodium channel shot-noise.

### Mutual information, I(Λ;TTS)

Treating the neuron as an information channel and as performing an experiment that estimates the value of Λ [61], the input-output variables are Λ and TTS, respectively. A proper calculation requires an appropriate range for both of these random variables. As in the fit to the histogram (Fig 4), the assumed range of TTS is the positive real line; however, as one can see in Fig 4, the probability of long and short durations is miniscule. Even though the earlier results demonstrate an expanded range for the intensities of synaptic activation beyond those reported by Hu et al, these ranges seem overly modest for a pyramidal neuron of neocortex whose average activity is under 10 Hz, implying the existence of firing times between a pair of pulses can be more than 100 ms. Regardless of the biophysical shortcomings of the model, an appropriate approximation for a meaningful mutual information requires one to extend the range of synaptic intensities beyond those values appearing in Fig 1. Therefore, we extended the range on the low-intensity end to 200 ms. In this case, the mutual information is *c.a*. 20% larger than the model with the lesser range. (see S1 Fig). The upper-bound of λ is also limited by the biophysical model. Here, without inhibition and with an average event of 200 pS, only limited increases of λ_*max*_ are possible. Such limited changes have a negligible effect on mutual information. For example, a 20% increase in λ_*max*_ increases mutual information from 3.0 bits/spike to 3.08 bits/spike, a 2.6% increase. Going in the other direction, a 25% decrease in λ_*max*_ causes a 4% decrease in bits/ spike.

Since we do not know the marginal probability distribution for Λ, we compare the result for three different distributions. The distributions, and each of their associated mutual information values, are summarized in Table 1. The calculations are described in the methods section.

**Table 1:** Distributions for Λ and associated mutual information values.

Distributional Form	Distribution	E[Λ] (events/ms)	Mutual Information (bits/spike)
*c*/λ (see [45])	1.07/λ	54.2	3.00
*c* (Uniform)	0.020	58.1	2.77
$\frac{\text{exp}(-\lambda /c)}{c\cdot [\text{exp}\left(-\lambda_{min}/c\right)-\text{exp}\left(-\lambda_{max}/c\right)]}$	$\frac{\text{exp}(-\lambda /100)}{28.59}$	55.9	2.99

Common to all distributions is the range of Λ: λ ∈ [32.8, 83.3] events/ms. For each distribution, there is a different value of *c* to achieve normalization.

## Discussion

Here, the discussion focuses on the following results: (i) discovery of a 1/TTS relationship consistent with a specific form of linear additivity and with a specific distributional form suggested by the linear additivity, (ii) the relative effects on mutual information of thermal noise, sodium channel shot-noise, and random synaptic activations, and (iii) mutual information, for which values here are compared to another in the literature.

### Linear additivity

Linear additivity is a pervasive assumption in many computational models, dating back to McCulloch and Pitts [1]; linear computation (or log-linear) is assumed, and seemingly required, for several recent computational models [44, 62, 63].

The results here show that, over a limited range of intensities, this biophysical model produces the desired inverse relationship between excitation intensity and TTS (Fig 1). Notably, this inverse proportionality occurs without manipulating the highly nonlinear biophysical parameters and specified localizations of the voltage-gated conductances, all of which were inherited from the consensus layer V pyramidal model [19, 46–49, 49, 51]. This inverse proportionality is observed for the nonstochastic, current-step injections at the soma (see S2 Fig), as well as observed for the stochastic, spatially distributed synaptic excitation. The somatic injection results suggest that the observed linearization generalizes to excitation distributed over basal dendrites, which connect to the soma. As expected, this extension is confirmed through a modest number of simulations.

Additionally, the model well approximates an inverse Gaussian (IG) distribution of the TTS (Fig 4). Indeed, this idea of the IG and the linearity required of the excitation process is quite old; a result first pointed out in neuroscience by Gerstein and Mandelbrot [2]. This linearization result is only approximately available to an Ornstein-Uhlenbeck diffusion because the approximation is good only at short time intervals [64] while the inverse linearization here is over a range of four time constants (*τ* = 12 ms). Empirically, at least two studies use the IG first hitting-time distribution to fit interpulse intervals using extracellular recording from intact animals [65, 66]. Moreover, one suspects the paucity of such IG fits is due to lack of trying rather than the failure of the fits themselves.

In addition to the existence of an empirically relevant biophysical model that produces the IG distributions of IPIs, such an IG fit supports the perspective of computational models that assume linear additivity of synaptic activation. That is, even though we know that the underlying biophysical processes are highly nonlinear and that this is a leaky neuron, the fits here are close to an IG. Therefore, the accumulation of internal excitation can be treated as linear additivity in the sense that an uncorrelated linearly additive continuous process hitting a straight barrier (nominal threshold) must be an IG [2, 56, 67].

On the other hand, supralinear summation has been observed neurophysiologically [31, 32]. But even in this case, the ideas advanced here may turn out to be applicable. Supralinear additivity, *e*.*g*. a time-independent but barrier-dependent interaction with voltage, can generate other first hitting-time distributions. Because first hitting-time is inversely proportional to input intensity for any member of the generalized IG class with the variance of synaptic events proportional to the intensity of events, these distributions are viable candidates for fitting models with supralinear excitation (see S1 Text for further explication). Unfortunately, in our hands, distinguishing between data-fits for appropriately parameterized IG vs. generalized IG distributions is problematic.

Studies suggesting sublinear charging of the initial segment are not consistent with our observations. A purely passive neuron (no voltage-activated channels) is a popular sublinear model that produces an Ornstein-Uhlenbech diffusion [6–8]. Although such models are unassailable for a passive neuron, the assumption of a purely passive neuron is somewhat dated.

In sum, the observations here serve as: (1) a prime example of complexity—the voltage-activated conductances—leading to simplicity—producing the equivalent of linear additivity of synaptic events and a closed-form probability distribution of first hitting-time; and (2) for computational models that assume IPI coding, the assumption of linear additivity is good enough, at least in terms of agreeing with a physiologically relevant, biophysical model of a neocortical neuron. In this regard, the observations of linear additivity *in vivo* [21, 26, 27] gives additional relevance to the findings here.

### Sources of noise

Some recent reviews have speculated on various qualitative sources of noise [64, 68–72] that would lower the information throughput of a neuron. It is fairly well-established that random synaptic arrivals (alternatively, synaptic noise) dominates [73] and that the second-largest source of unpredictable fluctuations is ion-channel shot-noise [74–76] (as noted in [68]). In this regard, voltage-activated sodium channels are believed to be the major contributor to total channel shot-noise [77, 78]. Here, we are concerned with a precise, quantitative statement of noise in the context of the initial segment of a consensus pyramidal neuron. Historically, perhaps the earliest relevant measurement estimating noise sources is [79]; they suggest that synaptic noise can account for all the variability in IPIs. Because their observations were performed *in vivo*, their results cannot directly isolate the noise contributions from the synaptic input versus channel noise; however, the modeling-based observations here are able to do so (Fig 3). Even more recent results [73] do not contradict the idea that synaptic noise dominates over channel-noise. Although their results suggest non-Poisson inputs, their interpretations are consistent with synaptic variability dominating VAC shot-noise variability in the physiological situation. Comparing the empirical, neurophysiological study to the biophysical model here, there is a notable difference in the duration of the excitatory inputs. In the empirical study, long-duration stimuli are used (lasting about one second) while here durations are used on the timescale of physiological IPIs. Apparently, the general conclusion of noise contributions is robust across such large differences in stimuli.

Under a very different set of assumptions, Sengupta et al. [80] also conclude that VAC shot-noise is only a minor and possibly ignorable source of input-output variability. Two distinctions stand out when comparing their results to the current study. Their model neuron is a biophysical neuron but seemingly somewhat arbitrary in its construction relative to the one studied here. Second, they model a dynamic input signal, perhaps inspired by the fly eye research. In contrast, the neuron of interest here can be thought of as performing computation (perhaps discrimination) over the interval of a visual fixation. In any case, the qualitative conclusions are the same.

While it is certainly true that noise limits the detection by peripheral sensors (hair cells in the cochlea, cones in the eye, etc.), we speculate that randomization processes are of much more concern in calculating information transmitted between neurons than explicit noise processes including thermal noise and shot noise of voltage-activated conductances. Rather than calling the unpredictable firing of a neuron ‘noise’, we prefer the term randomization, which is produced by the large number of poorly synchronized, input-line activations. Moreover, any such randomization process is itself enhanced by quantal synaptic failures [81].

### Mutual information

For the scene analysis problem considered here, one can propose the existence of a scalar, latent random variable, Λ, and a conjectured, continuous output code [14]. With these two hypotheses, the computational and information-transmission problem concerns estimating and communicating Λ = λ [45]. The input variable, λ, is a Poisson rate created by a union of the input lines, each treated as no more than a point process [82]. Thus, we assume a Poisson process as the input in calculating Shannon’s mutual information.

In any calculation of mutual information, one must assume an input distribution. Our assumption arises from a model inspired by pyramidal neuron firing in V1 during a visual fixation. For this model, most of the neurons, most of the time are firing well below their maximum firing rate. Of course, transiently some neurons are receiving an input close to their best input, in which case they would be driven for 2 or 3 pulses at 200 Hz [83, 84]. The study here allows for an approximation of this range with our defined priors. Since we do not know the correct prior distribution, we evaluate three possibilities, each of which is compatible with the dynamic range of the biophysical neuron being studied. Two of these prior probability distributions (the λ^−1^ distribution and the exponential distribution) reflect the fact that the neuron hardly spends any time firing at high frequencies.

Our estimate of mutual information agrees with the approximately 3 bits per spike calculated in [85]. This distinguished calculation of mutual information uses the fly H1 model to indirectly evaluate the effect of intrinsic noise under the assumption of a continuously and rather rapidly fluctuating visual scene (at the very least, 25 Hz, and certainly with higher-order frequency components present in the visual input). Such an input is consistent with the visual stimulus of a moving fly. However, for the visual fixation problem posited here, the driving input is, on average, changing at a rate less than 10 Hz. Nevertheless, the comparative values of bit rate are, perhaps surprisingly, in agreement. More broadly, the results here also agree with a range of other estimates, from 2 – 5 bits per spike [86–93]; see [94] for a review.

It can be argued that the 3 bits calculated here are an underestimate due to the constraints imposed on the consensus neuron being used. For example, the model considered here sustains firing only over a limited range of input intensities, and a more accurate neuron with more voltage-activated conductances (*e.g*. the *I*_*h*_ conductance or even a large set of spatially-distributed conductances [95, 96]) might lead to a neuron that has a greater dynamic range. Another limitation of the current study is a lack of inhibition. Physiologically, a neocortical neuron will simultaneously receive inhibition and excitation. Such inhibition downgrades the effect of synaptic events, which in turn requires larger values of Λ to produce the same firing rates. By increasing the number of synaptic events per output spike, the information rate per output spike increases. For example, a four-fold increase in the number of events needed to fire the neuron increases the mutual information by about 1 bit / spike. On the other hand, the conditional independence assumption (see Methods) might lead to a value that is inappropiately high.

### Suggestions for future research

Suggestions for future research include both biophysical modeling and experimental neurophysiology. The biophysical modeling should detail the interactions, as a function of both time and voltage, between the Na^+^ and K^+^ channels underlying the linearized relationship investigated here. In another direction, the biophysical models should also consider adding other conductances, particularly I_h_ (and novel ideas about this conductance [96]). Perhaps most challenging will be incorporation of well-quantified dendritic spiking phenomena. Regarding this challenge, there are at least two perspectives that require separate consideration: (i) analysis of the far apical spike with regard to 1/TTS and (ii) the analysis of the union of (a) the basal dendritic spikes and (b) the spikes of the near apical dendritic branches.

Regarding empirical intracellular research, there are again two suggestions using a layer V neocortical pyramidal neuron. First, using the TTS observations as before, there is a need to extend the intensity range studied to include both higher and lower intensities leading to both shorter and longer TTSs than currently available. Second, the observations and inferences here also motivate twin-pulse excitations; that is, how does TTS vary as a function of the time since the most recent output spike? Finally, we look forward to basic TTS observations for layer 2/3 pyramidal neurons.

The work here is no more than a first step in characterizing functionality consistent with linear additivity; for example, it is restricted to only one type of neuron and the available neurophysiological observations [46, 47, 51, 52]. Whether the inverse relationship between TTS and input intensity, and therefore the viability of the linear additivity hypothesis, will occur in other pyramidal neurons remains an open question. With the advent of new constraining neurophysiological data to go along with a more detailed knowledge of the voltage-activated channels, the hypotheses raised here can be retested and modified as necessary. Hopefully, the way we have analyzed the biophysical data here will encourage neurophysiologists to measure and analyze their empirical data in a similar manner.

## Methods

### Model

This work models a Layer 5 pyramidal cell quantified by Mainen and Sejnowski [47] (their Fig 1D). The biophysical model evolved from [51] in which it was used to study back propagating action potential spikes. This led to [46] in which it was further refined (model can be retrieved from ModelDB [97]). The model was fit to measurements at several different places on the neuron including the AIS, axon, and dendrites.

The model includes both Nav 1.6 and Nav 1.2 channels distributed along the AIS. It is well-accepted that the action potential is initiated at the distal AIS [52, 54, 98] with its high concentration of both sodium channel types [53, 55, 99]. The parameter sweeps of [46] (see their Fig 5) severely restrict the placement of the two types of sodium channels. The capacitivity in the soma is changed from 1 μF/cm^2^ to 0.9 μF/cm^2^, as this is a more realistic value [100]. In addition, a small amount of persistent Na channels is added at the proximal apical dendrite to simulate more realistic neuronal firing over a longer range of times. The persistent sodium channels are modelled using the Nav1.6 channels with shifted inactivation rate equations, so that they would inactivate much more slowly [59, 98, 101].

The main change is the alteration of sodium channels so that they operated stochastically. The stochastic sodium channels are modeled with a widely-used eight-state kinetic reaction scheme describing the *m*^3^*h* Hodgkin-Huxley activation kinetics [102, 103] quantified in [50]. This gating scheme is shown in [104] (their Fig 4A). When run deterministically, there is no variability. These changes do not noticeably change the overall shape of the action potential (Fig 5). The main difference is that the stochastic voltage-trace lies just below the deterministic trace 200–300 μs before firing. Since it is noisier, the stochastic neuron can suddenly cross threshold with a stochastic event, and thus does not consistently approach threshold in the gradual manner of the deterministic neuron. Thus, compared to the deterministic neuron, the stochastic neuron tends to be at a slightly lower voltage when it fires (see S4 Fig).

### Excitation

Simulations are performed using the simulation environment NEURON [105]. All simulations are run at 37°C with a time step of 1 μs, and the results do not change for smaller time steps. In all cases, the neuron is allowed to come to steady state before the stimulus is applied. The neuron is stimulated in the main apical dendrite 250 μm from the soma. For these layer V pyramidal cells, our interests center on synapses that exclude the distal tuft which we presume has its own independent computations (for example see [44, 106, 107]). Then, noting the anatomy of such layer V cells (see Fig 1A in [108] or Fig 1d in [47]), the vast majority of dendritic surface area, and therefore synaptic localizations, is within 300 μm of the cell body. That is, the cell’s dendritic surface area is dominated by basal and near-apical dendritic branches whose distal tips are almost inevitably less than 300 μm from the cell body.

Stimulation is performed in two ways: first with a noise-free current-step in the main apical dendrite, and second by simulating synaptic activity. Synaptic activity is simulated using synapses distributed evenly along the main apical dendrite 200 μm-300 μm from the soma. A small number of simulations are performed by scattering synapses on five of the basal dendrites with no differences noted from the main set of simulations. Synapses are simulated as the Poisson arrival of square pulses of 200 pS lasting 1.2 ms with a reversal potential of 0 mV. The Poisson assumption used here arises not from a Poisson assumption on individual inputs, but the Poisson approximation [109] produced by the unioning of all the input lines, each one being a point process.

The restricted range of current-step intensities begin with those of the physiological studies of Hu et al. 2009; that is, their range is the starting point upon which we expand. At very high intensities, their model tends to initiate spiking from the near apical dendrite. The physiological nature of such a dendritically originated spike remains an open question. Because there are such strong arguments for initial segment initiations [52], we postpone using this biophysical observation until neurophysiology confirms or denies its existence.

### Mutual information calculation

Treating the neuron as an information channel, we identify the input random variable as Λ and the output random variable as TTS. In order to calculate the mutual information *I*(Λ;*TTS*), we require the pair of distributional forms *P*(Λ) and *P*(*TTS*|Λ = λ) for all λ. *P*(*TTS*) is inferred from these forms (see Eq 2) and then calculate the mutual information (see Eq 4).

#### P(Λ)

Three distinct distributions are investigated for *P*(Λ). In all cases, these distributions were assumed to have the finite range of interest here. Very low intensities of excitation that never yield a spike are of no interest because there is no IPI to decode. Likewise, extremely high intensity input, as is possible with intracellular current injection but not possible using synaptic input, is out of the range of interest. Finally, any aspects of the neuron’s voltage once threshold is closely approached and achieved is not of interest. See Table 1 for the investigated distributional forms and the associated range of Λ.

#### P(TTS|Λ)

The results suggest that at a given intensity λ, the distribution of TTS is well-approximated by an inverse Gaussian distribution (see Fig 2). The PDF of the inverse Gaussian distribution with a mean of *μ* and a shape parameter of *ρ* is (here tts is a realization of the random variable TTS):

$$\begin{array}{c} IG\left(\mu \left(\lambda \right),\rho \left(\lambda \right)\right)={\left(\frac{\rho (\lambda )}{2\pi tts^{3}}\right)}^{1/2}\;exp\;(\frac{{-\rho \left(\lambda \right)tts-\mu \left(\lambda \right))}^{2}}{2\mu {\left(\lambda \right)}^{2}tts}) \end{array}$$ (1)

An expression for *P*(*TTS*|Λ) requires the parameters *μ* and *ρ* for any λ in the assumed range. Given a value of λ and eventually using sample values for population values, the mean *μ*(λ) corresponds to *E*[*TTS*|λ] and the shape parameter *ρ*(λ) corresponds to *E*[*TTS*|λ]^3^/*Var*[*TTS*|λ]. The conditional sample mean, *c*.*a*. *E*[*TTS*|λ], is approximated with a best-fit to the collected data plotting *E*[*TTS*|λ] as a function of 1/λ, and the conditional sample variance, *c*.*a*. *Var*[*TTS*|λ], is approximated with a best-fit to the collected data plotting *Var*[*TTS*|λ] as a function of 1/λ. When it was necessary to extrapolate the variance beyond the range of collected data, the largest observed variance was used (18.57 ms^2^). The regression generated means and variances are in S1 Table.

#### P(TTS)

*P*(*TTS*) is computed by numerical integration with Mathematica over the entire range under consideration.

$$\begin{array}{c} P\left(TTS\right)=\int_{\lambda =32.8\;\text{events/ms}}^{\lambda =83.3\;\text{events/ms}}\;P\left(TTS|\Lambda =\lambda \right)\cdot P_{\Lambda }\left(\lambda \right)d\lambda \end{array}$$ (2)

The resulting *P*(*TTS*) distributions are shown in S3 Fig.

#### I(Λ;TTS)

The mutual information (in bits / spike) is given by the following double integral.

$$\begin{array}{c} I\left(\Lambda ;TTS\right)=\int_{\lambda =32.8\;\text{events/ms}}^{\lambda =83.3\;\text{events/ms}}\;P_{\Lambda }\left(\lambda \right)\int_{0}^{\infty }\;P\left(TTS|\lambda \right)log_{2}\frac{P(TTS|\Lambda =\lambda )}{P(TTS)}dttsd\lambda \end{array}$$ (3)

This double integral is approximated using the following equation by summing over Λ with steps of 0.1 events/ms and by summing over TTS in the range [1 ms, 250 ms] with steps of 0.05 ms. Decreasing this step size by half does not change the value of the summation.

$$\begin{array}{c} I\left(\Lambda ;TTS\right)=\sum_{\lambda =32.8\;\text{events/ms}}^{\lambda =83.3\;\text{events/ms}}\;P_{\Lambda }\left(\lambda \right)\sum_{1\;\text{ms}}^{250\;\text{ms}}\;P\left(TTS|\lambda \right)log_{2}\frac{P(TTS|\Lambda =\lambda )}{P(TTS)} \end{array}$$ (4)

The mutual information calculation assumes independence between successive values of the latent variables.

## Supporting information

S1 Fig Mutual information values over different extrapolated ranges. All calculations share the same shortest TTS (6.8 ms) and use the same distributional form for Λ (c/λ, where c is a constant). The maximum value of λ is 83.33 events/ms. The minimum value of λ changes depending upon the longest TTS in the range, and the value of c is chosen to normalize the distribution.
(EPS)

S2 Fig A linear inverse TTS as a function of somatic excitation. Excitation is a point current-step in the soma. The dashed line is a best linear fit. Each point is an average of 120 excitations from rest. The error bars (SEM) for the current-step are within the plot points. All points have spike initiation at the AIS.
(EPS)

S1 Text (PDF)

S1 Table Values for discrete intensity steps and the corresponding sample statistics. (PDF)

S3 Fig *P(TTS)* distributions. (A) The *P*(*TTS*) distribution (thick blue curve) is computed as the weighted sum of conditional distributions. Conditional distributions, *P*(*TTS*|λ), are shown for λ = 80 events/ms (red dashed line), λ = 50 events/ms (green dotted line), and λ = 40 events/ms (thin black line). (B) The resulting discrete *P*(*TTS*) distributions (approximating the continuous densities) implied by the three different Λ distributions of Table 1.
(EPS)

S4 Fig Voltage traces responding to a current-step and stochastic synaptic-step. Voltage is measured at the AIS. The current-step is 0.7 nA, and the stochastic synaptic step is 58.33 events/ms. Although the average input current is the same, the action potential evoked by the stochastic conductance step is, in general, later than the action potential evoked by the current step (see histograms in Fig 5) This delay occurs because the synaptic conductance events are distributed over a 100 μm section of the apical dendrite while the current-step is a point source in this dendrite.
(EPS)
