# Supplementary material for: A consensus layer V pyramidal neuron can sustain interpulse-interval coding
Source: PLoS One. 2017 Jul 13;12(7):e0180839. doi: 10.1371/journal.pone.0180839 (PMC5509228; doi:10.1371/journal.pone.0180839)
Supplement: S1 Text — (PDF) [file pone.0180839.s003.pdf]

The results here do not mandate that the net effect of synaptic excitation must appear as linearly additive to the neurophysiologist. This hypothesis is inspired by [1] and the examples there. Although the linearly additive model produces the desired results, there are also nonlinear models that produce the desired inverse relationship $E[1/t|\lambda] \propto \lambda$, where λ is the synaptic input intensity and t is the time-to-spike. All of these nonlinear diffusion models produce the inverse proportionality observed here; all the ones we know about require that the rate of variance increase, with time, is in direct proportion to the excitation rate itself, λ; and all of these distributions are first hitting times in the generalized IG family [1]. They take the form $P(t|\lambda) = t^{a-1}exp(\frac{-b_1\lambda t}{2} - \frac{b_2}{2\lambda t})/(2\left(\frac{b_1\lambda^2}{b_2}\right)^{-a/2} K_a(\sqrt{b_1 b_2}))$ where $K()$ is a modified Bessel function of the second kind and a defines the exact member of the family. For any member of this family, calculating the mean of inverse time-to-hit (using Mathematica) yields $E[T^{-1}|\lambda] = \lambda\frac{\sqrt{b_1}K_{a-1}(\sqrt{b_1 b_2})}{\sqrt{b_2}K_a(\sqrt{b_1 b_2})} \propto \lambda$, *i.e.*, the desired inverse relationship (the Bessel functions can be treated as part of the proportionality constant because they do not contain any terms of λ).

To create other models that are not IG but that are generalized IG, note the examples in [1]. These examples illustrate distance dependent interactions with the barrier. Our speculative neuroscientific interpretation takes distance to be the difference between membrane potential at the spike initiation site and nominal threshold. A supralinear effect corresponds to a barrier that is increasingly attractive as distance decreases. The model even allows a certain kind of sublinearity arising from a mildly repulsive barrier, with repulsion increasing as distance decreases. Again speculatively, extra voltage-activated Na^+ channels create an attractive barrier while extra voltage-activated K^+ channels create repulsion.

[1] Barndorff-Nielsen O, Blsild P, Halgreen C. First hitting time models for the719generalized inverse Gaussian distribution. Stochastic Processes and their720Applications. 1978;7(1):4954.
