# Supplementary material for: A consensus layer V pyramidal neuron can sustain interpulse-interval coding
Source: PLoS One. 2017 Jul 13;12(7):e0180839. doi: 10.1371/journal.pone.0180839 (PMC5509228; doi:10.1371/journal.pone.0180839)
Supplement: S1 Table — (PDF) [file pone.0180839.s004.pdf]

	Step Intensity (nA / events/ms)	E[TTS] (ms)	Var[TTS] (ms^2)	S.D.[TTS] (ms)
**Stochastic Synaptic Step**	83.33	6.86	0.52	0.72
	70.83	9.44	0.44	0.66
	58.33	13.14	0.91	0.96
	55.83	14.29	1.20	1.09
	54.17	15.25	1.52	1.23
	51.67	17.14	2.66	1.63
	50.00	18.83	4.05	2.01
	49.17	19.99	5.20	2.28
	48.75	20.64	5.48	2.34
	48.33	21.35	7.53	2.74
	47.50	22.99	10.05	3.17
	47.08	24.28	18.57	4.31
**Current Step**	1	5.44	0.002	0.04
	0.85	8.02	0.005	0.07
	0.7	12.20	0.012	0.11
	0.67	13.37	0.024	0.16
	0.62	15.98	0.025	0.16
	0.58	19.27	0.078	0.28
	0.55	23.30	0.138	0.37
	0.525	29.34	0.454	0.67
